# Aerobic Exercise Inhibits Sympathetic Nerve Sprouting and Restores β-Adrenergic Receptor Balance in Rats with Myocardial Infarction

**DOI:** 10.1371/journal.pone.0097810

**Published:** 2014-05-19

**Authors:** Ting Chen, Meng-Xin Cai, You-You Li, Zhi-Xiong He, Xiu-Chao Shi, Wei Song, You-Hua Wang, Yue Xi, Yu-Ming Kang, Zhen-Jun Tian

**Affiliations:** 1 Institute of Sports and Exercise Biology, Shaanxi Normal University, Xi’an, Shaanxi, P. R. China; 2 Department of Sports and Exercise, Tibet University for Nationalities, Xian yang, Shaanxi, P. R. China; 3 Department of Physiology and Department of Cardiology, Fourth Military Medical University, Xi’an, Shaanxi, P. R. China; 4 Department of Physiology and Pathophysiology, Xi’an Jiaotong University Cardiovascular Research Center, Xi’an Jiaotong University School of Medicine, Xi’an, Shaanxi, P. R. China; Max-Delbrück Center for Molecular Medicine (MDC), Germany

## Abstract

**Background:**

Cardiac sympathetic nerve sprouting and the dysregulation of β-adrenergic receptor (β-AR) play a critical role in the deterioration of cardiac function after myocardial infarction (MI). Growing evidence indicates that exercise provides protection against MI. The aims of this study were to investigate whether aerobic exercise following MI could inhibit sympathetic nerve sprouting and restore the balance of β3-AR/β1-AR.

**Methods:**

Male Sprague-Dawley rats were divided into three groups: sham-operated control group (SC), MI group (MI), and MI with aerobic exercise group (ME). The rats in ME group were assigned to 8 weeks of exercise protocol (16 m/min, 50 min/d, 5 d/wk). The expression of nerve growth factor (NGF), the sympathetic nerve marker-tyrosine hydroxylase (TH), the nerve sprouting marker-growth associated protein 43 (GAP43), and β1- and β2-AR expression in the peri-infarct area of the left ventricle (LV) were measured by Western blot and immunohistochemistry, while β3-AR expression was determined by Western blot and immunofluorescence. Endothelial nitric oxide synthase (NOS2), phospho-NOS2 (p-NOS2), and neuronal nitric oxide synthase (NOS1) were measured by Western blot.

**Results:**

MI increased LV end-diastolic pressure (LVEDP), and decreased LV systolic pressure (LVSP). Compared with the MI group, aerobic exercise significantly decreased LVEDP and increased LVSP. The protein expression of TH, GAP43 and NGF was significantly increased after MI, which was normalized by exercise. Compared with the SC group, the ratios of β2-AR/β1-AR and β3-AR/β1-AR were elevated in the MI group, and the protein expression of β3-AR and NOS1 increased after MI. Compared with the MI group, the ratios of β2-AR/β1-AR and β3-AR/β1-AR were normalized in the ME group, while the protein expression of β3-AR and NOS1 significantly increased, and NOS2 was activated by exercise.

**Conclusions:**

Aerobic exercise inhibits cardiac sympathetic nerve sprouting, restores β3-AR/β1-AR balance and increases β3-AR expression through the activation of NOS2 and NOS1 after myocardial infarction.

## Introduction

Myocardial infarction (MI) induces cardiac sympathetic nerve sprouting in humans [1] and in animal models [2]. Cardiac nerve growth factor (NGF) is a major neurotrophin correlated with sympathetic nerve sprouting [3], and NGF plays an important role in synapse formation and axonal growth during sympathetic neuron development [4]. Zhou et al. [2] demonstrated that MI increased cardiac NGF and growth associated protein 43 (GAP43) expression in the infarcted area. NGF and GAP43 were then transported retrogradely to the left stellate ganglion, which resulted in sympathetic nerve sprouting in the noninfarcted area [2], [5]. However, excessive nerve sprouting suppressed the functions of transient outward current and inward rectifier current [6], thereby increasing the susceptibility to ventricular arrhythmias [7], [8]. Accordingly, inhibition of sympathetic nerve sprouting processes may provide an effective therapy to prevent arrhythmias.

In the failing heart, the sustained sympathetic activation results in downregulation of the β1-adrenergic receptor (β1-AR) [9], desensitization of β1- and β2-AR, and upregulation of β3-AR [10]. The dysregulation of β-AR [9], particularly the opposite changes in β1- and β3-AR expression [10] plays a key role in left ventricular (LV) remodeling [11] and ventricular arrhythmias [12], [13]. Thus, restoration of the β-AR balance in the heart may result in improved cardiac function. Recently, β3-AR has been regarded as a protective factor in the development of MI [14]. The absence of β3-AR exacerbated cardiac adverse ventricular remodeling, enhanced oxidative stress and nitric oxide synthase (NOS) uncoupling [15], [16]. This beneficial effect of β3-AR was associated with endothelial nitric oxide synthase (NOS2) [17] and neuronal nitric oxide synthase (NOS1) activation [18]. However, the role of β3-AR in mediating the cardioprotective effects of exercise following MI remains unclear.

Exercise is an important clinical intervention for the prevention and treatment of MI [19]–[21]. It is well established that exercise decreases sympathetic activity after MI [22]–[24]. And in the diseased heart, exercise can increase β-AR density [9], increase β1-AR protein levels [25], and reduce β2-AR responsiveness [26]. Additionally, a more normal β1/β2-AR balance can be restored by exercise in animals susceptible to sudden death [26]. However, few studies have examined the effects of aerobic exercise on sympathetic nerve sprouting and β3-AR/β1-AR balance after MI. The aims of this study were to investigate whether aerobic exercise could inhibit sympathetic nerve sprouting and restore the balance of β3-AR/β1-AR, and to determine any role the β3-AR, NOS2 and NOS1 signaling pathways may play in the beneficial effects of exercise after MI.

## Methods

### Animals

Male Sprague-Dawley rats (204±6 g, 8-weeks old, n = 42) were provided by the Laboratory Animal Centre of Xi’an Jiaotong University. These studies were performed in accordance with the “Guiding principles for research involving animals and human beings” [27]. All experimental protocols were approved by the Review Committee for the Use of Human or Animal Subjects of Shaanxi Normal University.

### Surgical Procedure

MI was induced by ligation of the left anterior descending coronary artery under anesthesia (pentobarbital 30 mg/kg) as previously described [28]. The coronary artery was ligated approximately 2.0 mm from its origin using a 6.0 silk suture (MI rats, n = 30). Sham-operated rats (SC; n = 12), which underwent the operation without coronary artery ligation, served as a control group. A standard 12-lead electrocardiogram was used to document ST-segment elevation. Two rats died during the surgery and four rats died 2 h after surgery.

### Aerobic Exercise Protocol

Seven days after the surgery, MI rats (n = 24) were randomly divided into two groups: the MI group (MI, n = 12), and the MI with aerobic exercise group (ME, n = 12). Rats in the ME group were submitted to 8 weeks of aerobic exercise using a motorized rodent treadmill (DSPT-202, Li Tai Technology, Hangzhou, China), while the other group remained sedentary throughout the experiment period. To allow a gradual adaptation to the exercise stress, training was initiated at 10 m/min at a 5°incline for 10 min per day. During the second week, the speed and duration were gradually increased to 16 m/min and 50 min per day (including a 5-min warm-up at 10 m/min), which was maintained constant throughout the experiment. The training intensity was approximately 55% of maximal oxygen uptake (V_O2max_) [29], [30]. This exercise regimen was well tolerated by MI rats. There were no mortalities during the 8 weeks of aerobic exercise.

### Hemodynamic Measurement

At the end of the 8 weeks of training or sedentary behavior, rats were anesthetized as mentioned above. A pressure transducer was inserted retrograde from the right carotid artery to the LV cavity, and traditional intraventricular catheter recordings (Powerlab 8/30, ML 870, ADInstruments, Castle Hill, Australia) were performed to evaluate cardiac function. The following hemodynamic parameters were measured: LV systolic pressure (LVSP, mmHg), LV end-diastolic pressure (LVEDP, mmHg), maximal positive and negative first derivative of LV pressure (±dp/dt_max_), and the time constant of LV pressure decay (Tau). All rats were euthanized after hemodynamic measurements.

### Cardiac Morphometry

The infarct size was evaluated by triphenyltetrazolium chloride (TTC) staining. Briefly, the heart was cut into six transverse slices, and incubated for 30 min in a 1% TTC solution to differentiate the infarcted (pale) from viable (brick red) myocardial area. The total area of necrosis was calculated by planimetry using IMAGE-PRO PLUS 6.0 (IPP 6.0, Media Cybernetics, Bethesda, MD, USA) and expressed as percentage of the total LV area.

Heart samples taken from the LV infarct border area were fixed in ice-cold 4% paraformaldehyde for 24–48 h, embedded in paraffin and sectioned (5 µm thick) for histopathologic examination. The slices were stained with Masson’s trichrome, and were used to observe the construction of cardiac tissue in the infarct area of the LV. To evaluate the degree of fibrosis, the collagen volume fraction (CVF) was measured in 10 fields for each LV section of Masson’s trichrome staining. CVF (fibrosis area/total area of myocardium) values were calculated using IPP 6.0.

### Immunohistochemical (IHC) Staining

Briefly, the paraffin sections were incubated with the following diluted primary antibodies overnight at 4°C: TH (1∶400, Millipore, Billerica, MA, USA), GAP43 (1∶500, Millipore), NGF (1∶100, Signalway Antibody, Pearland, TX, USA). β1-AR (1∶150, Bioworld, Atlanta, Georgia, USA), and β2-AR (1∶150, Signalway Antibody). Following this incubation, sections were exposed to a secondary antibody for 30 min at room temperature. The stains were developed using diaminobenzidine as a chromogen. The sections were then counterstained with hematoxylin and examined by conventional light microscopy (Olympus BX51, Olympus Optical, Tokyo, Japan). Six sections from each group were scanned, with 10 fields per section viewed, and the value of mean optical density (OD) was calculated by IPP 6.0. The density of stained cardiac sympathetic nerves was determined using IPP 6.0 and expressed as the nerve area divided by the total area examined (um^2^/mm^2^). The nerve density of each slide was determined by the mean density of nerves calculated from all three selected fields.

### Immunofluorescence (IFC) Examination

The paraffin sections were incubated in the rabbit polyclonal antibody β3-AR (1∶50 dilution, Santa Cruz Biotechnology, Santa Cruz, CA, USA) overnight at 4°C. As a negative control, PBS was used in place of the primary antibody. Then TRITC-conjugated goat anti-rabbit IgG (1∶100 dilution, Jackson ImmunoResearch, West Grove, PA, USA) was used as the secondary antibody to detect the primary antigen-antibody reaction. The nuclei was stained by 4′-6-diamidino-2-phenylindole (DAPI) dye (1∶2000 dilution, Sigma, St Louis, MO, USA). Immunofluorescent labeling of the sections were observed with a fluorescence microscope (Nikon Eclipse 55i, Nikon, Tokyo, Japan). Quantification of the β3-AR fluorescence density was determined by IPP 6.0.

### Western Blotting (WB)

The tissues from the LV infarct border area (5 mm) was homogenized. Total proteins were extracted with RIPA lysis buffer containing protease inhibitors (Roche, Indianapolis, IN, USA). Protein samples were separated by sodium dodecyl sulphate-polyacrylamide gel electrophoresis and transferred to nitrocellulose membranes (Millipore, Billerica, MA, USA). The membrane was incubated with the following diluted primary antibodies: TH (1∶500, Millipore), GAP43 (1∶1000, Millipore), NGF (1∶500, Signalway Antibody), β1-AR (1∶2000, Bioworld), β2-AR (1∶1000, Signalway Antibody), β3-AR (1∶800, Santa Cruz), NOS2 (1∶500, Cell Signaling, Beverly, MA, USA), phosphorylation of NOS2 at serine residue 1177 (p-NOS2^ser1177^, 1∶500, Cell Signaling), NOS1 (1∶400, Signalway Antibody), at 4°C overnight. Following washing, the membrane was incubated with horseradish peroxidase-conjugated secondary antibodies (1∶10000 dilution, Jackson, ImmunoResearch, USA). GAPDH was used as an internal control. Protein bands were subsequently detected with enhancedchemiluminescence and sections were exposed to X-ray film.

### Statistical Analysis

All data in this study are expressed as the mean ± SD. Differences between mean values in the three groups were analyzed by one-way analysis of variance (ANOVA), followed by post-hoc testing using the Student-Newman-Keuls’ test when appropriate. The *F* values (with degrees of freedom) were included. *P*<0.05 was considered significant.

## Results

### Aerobic Exercise Reduces Infarct Size and Myocardial Interstitial Fibrosis after MI

**Figure 1:**
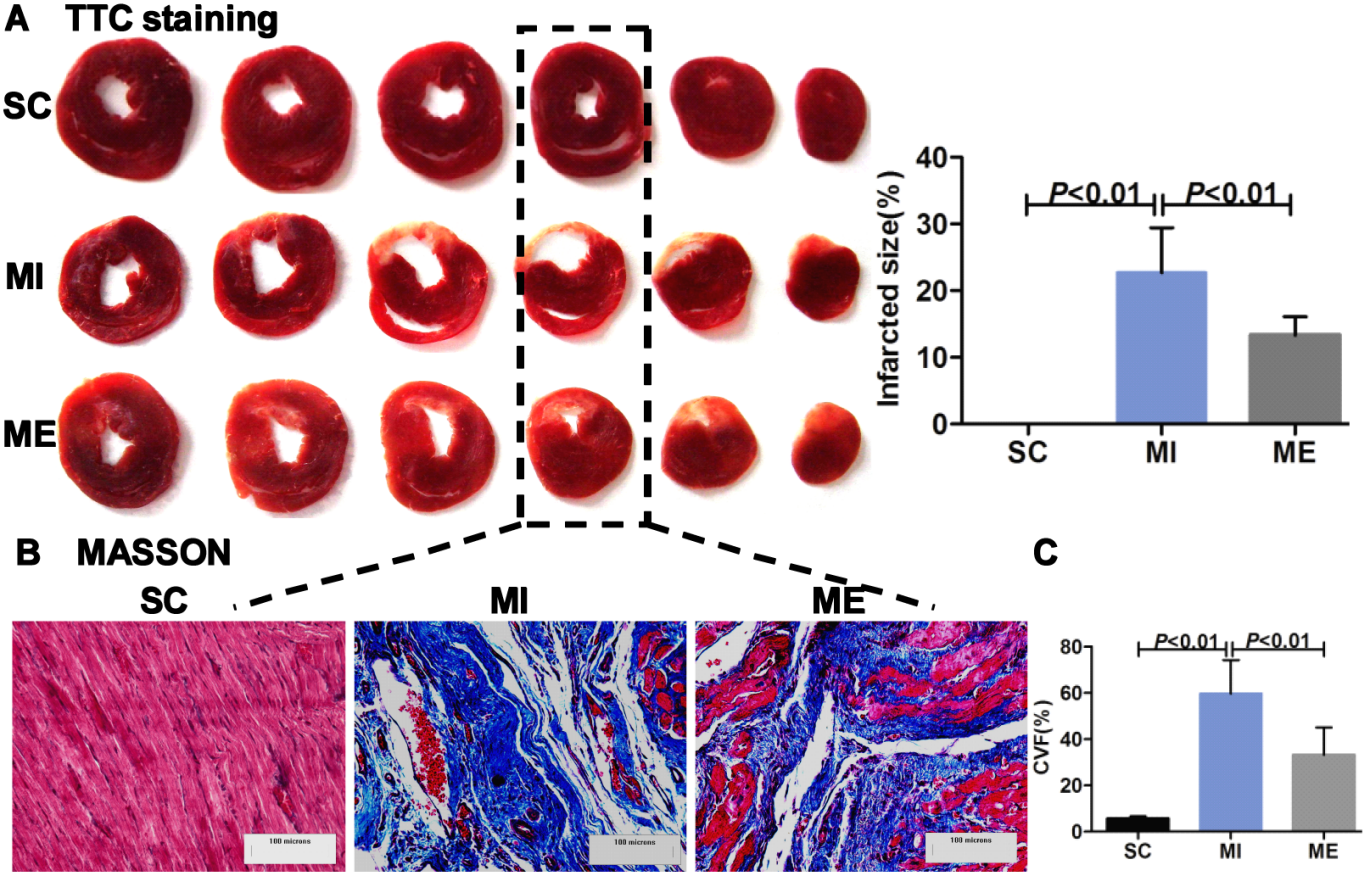
Effects of aerobic exercise on infarct size and cardiac structure after MI. A. Representative triphenyltetrazolium chloride staining images. Infarct size was significantly attenuated in the ME group compared with the MI group. B. Masson’s trichrome staining. Myocardial fibrosis was significantly increased in rats in the MI group compared with the SC and ME groups. Scale bar = 100 microns. C. Collagen volume fraction (CVF) in the left ventricle of rats in the SC, MI, ME group. Quantitative analysis showed that CVF was significantly higher in the MI group compared with the SC and ME groups. The values are expressed as means ± SD (n = 6). SC, sham-operated control group. MI, MI group. ME, aerobic exercise with the MI group.

The result of TTC staining showed that the infarct area was significantly reduced in the ME group (13.32±2.77%) compared with the MI group (22.69±6.71%, *P*<0.01) (**Figure 1A**).

As shown in Figure 1B, in the MI group, the cardiac structure was disordered with extensive fibrotic tissue (blue staining) compared with the SC group. After 8 weeks of aerobic exercise, there was a trend towards reduction in cardiac fibrosis compared with the MI group (**Figure 1B**). CVF (*F*_2,16_ = 23.19, *P*<0.001), an indicator of interstitial fibrosis, was significantly higher in the MI group (59.64±4.83%, *P*<0.01) than in the SC group (5.79±0.52%). Aerobic exercise (33.23±4.44%, ME group, *P*<0.01) significantly reduced CVF compared with the MI group (**Figure 1C**).

### Aerobic Exercise Attenuates the Deterioration in Cardiac Function after MI

As illustrated in Table 1, the changes of hemodynamic parameters following MI indicated a severe cardiac dysfunction, as evidenced by a significant increase in LVEDP (*F*_2,33_ = 8.55, *P* = 0.002) and Tau (*F*_2,33_ = 4.12, *P* = 0.022) (both *P*<0.01), and a decrease in LVSP (*F*_2,33_ = 5.77, *P* = 0.006) and ±dp/dtmax (*F*_2,33_ = 8.36, *P* = 0.001. *F*_2,33_ = 8.73, *P* = 0.001, respectively) (both *P*<0.01) compared with the SC group. And MI was recognized on an electrocardiogram by ST-segment elevation (**Figure S1**). However, 8 weeks of aerobic exercise resulted in a significant reduction in LVEDP and Tau (both *P*<0.01), and an increase in LVSP and LV±dp/dtmax (both *P*<0.01) compared with the MI group (**Table 1**).

**Table 1:** Effects of aerobic exercise on hemodynamic parameters in rats.

	SC	MI	ME
LVSP (mmHg)	112.47±8.63	104.62±8.19**	118.92±8.97##
LVEDP (mmHg)	2.66±0.44	9.67±1.05**	2.53±0.58##
+dP/dt (mmHg/s)	5363.54±315.40	3777.79±907.99**	5074.38±784.99##
–dP/dt (mmHg/s)	4036.22±912.60	2482.18±724.77**	4079.15±510.52##
Tau (ms)	19.37±3.97	27.37±6.75**	20.02±3.67##

Values are expressed as means ± SD. n = 8 in each group. SC, sham-operated control group; MI, MI group; ME, aerobic exercise with MI group.

Left ventricular systolic pressure (LVSP), LV end-diastolic pressure (LVEDP), positive and negative maximum values of the instantaneous first derivative of LV pressure (±dp/dtmax), the time constant of left ventricular pressure decay (Tau).

*P<0.05,

**P<0.01vs. SC group;

# *P*<0.05,

## *P*<0.01 vs. MI group.

### Aerobic Exercise Inhibits Cardiac Sympathetic Nerve Sprouting after MI

**Figure 2:**
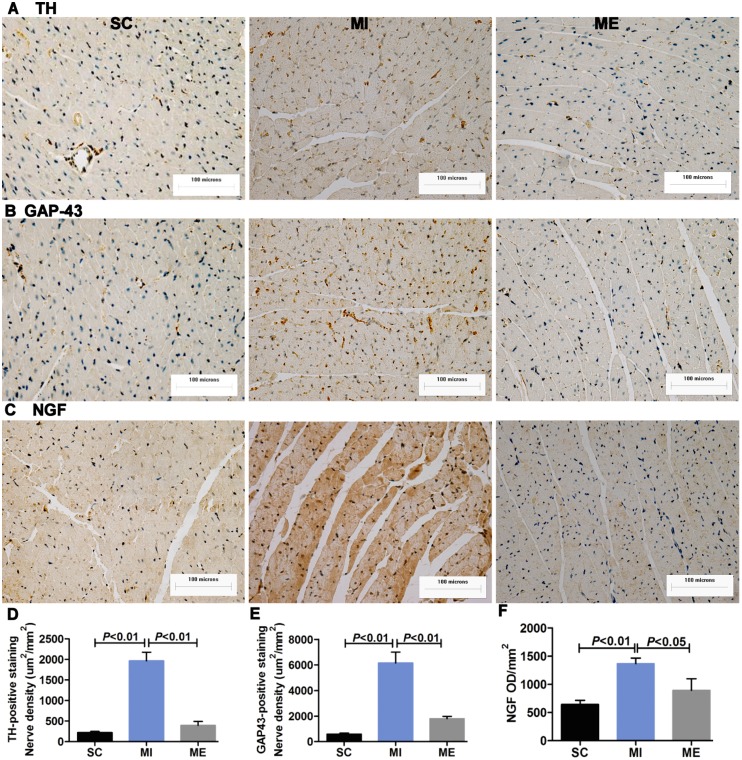
Immunohistochemical staining of cardiac TH, GAP43 and NGF. A–C. Immunohistochemical staining of cardiac TH, GAP43 and NGF. Scale bar = 100 microns. D–F. The mean optical density value of cardiac TH, GAP43 and NGF. Immunohistochemical staining showed that cardiac TH, GAP43 and NGF protein expression significantly increased after MI, which was normalized by aerobic exercise.

**Figure 3:**
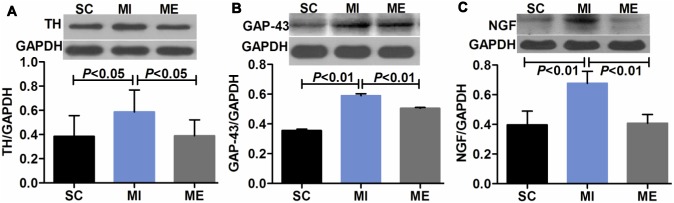
Western blot analysis of cardiac TH, GAP43 and NGF proteins. A–C. Western blot analysis of cardiac TH, GAP43 and NGF proteins in the SC, MI, ME groups. The GADPH level was used as an internal control. Western blot analysis showed that cardiac TH, GAP43 and NGF protein expression significantly increased after MI, which was normalized by aerobic exercise.

IHC staining showed that compared with the SC group, abundant TH- (*F*_2,15_ = 57.36, *P*<0.001) and GAP43- (*F*_2,15_ = 31.17, *P*<0.001) positive nerves were observed in the LV after MI (both *P*<0.01), but the densities of TH- and GAP43-positive nerves were significantly decreased (both *P*<0.01 vs. MI) by aerobic exercise (**Figure 2A, B, D, E**). Compared with the SC group, the mean OD value for NGF (*F*_2,16_ = 10.96, *P*<0.001) in the LV increased after MI (*P*<0.01). However, following 8 weeks of aerobic exercise, the value significantly decreased compared with the MI group (*P*<0.05) (**Figure 2C, F**). As shown in Figure 3A, B, C, compared with the SC group, the protein expression of TH (*F*_2,15_ = 3.01, *P* = 0.08), GAP43 (*F*_2,12_ = 9.38, *P* = 0.004) and NGF (*F*_2,16_ = 45.11, *P*<0.001) in the LV was significantly increased after MI (*P*<0.05, *P*<0.01, and *P*<0.01, respectively), which was normalized by aerobic exercise (*P*<0.05, *P*<0.01, and *P*<0.01, respectively) (**Figure 3A, B, C**).

### Aerobic Exercise Restores Cardiac β-adrenergic Receptor Balance after MI

**Figure 4:**
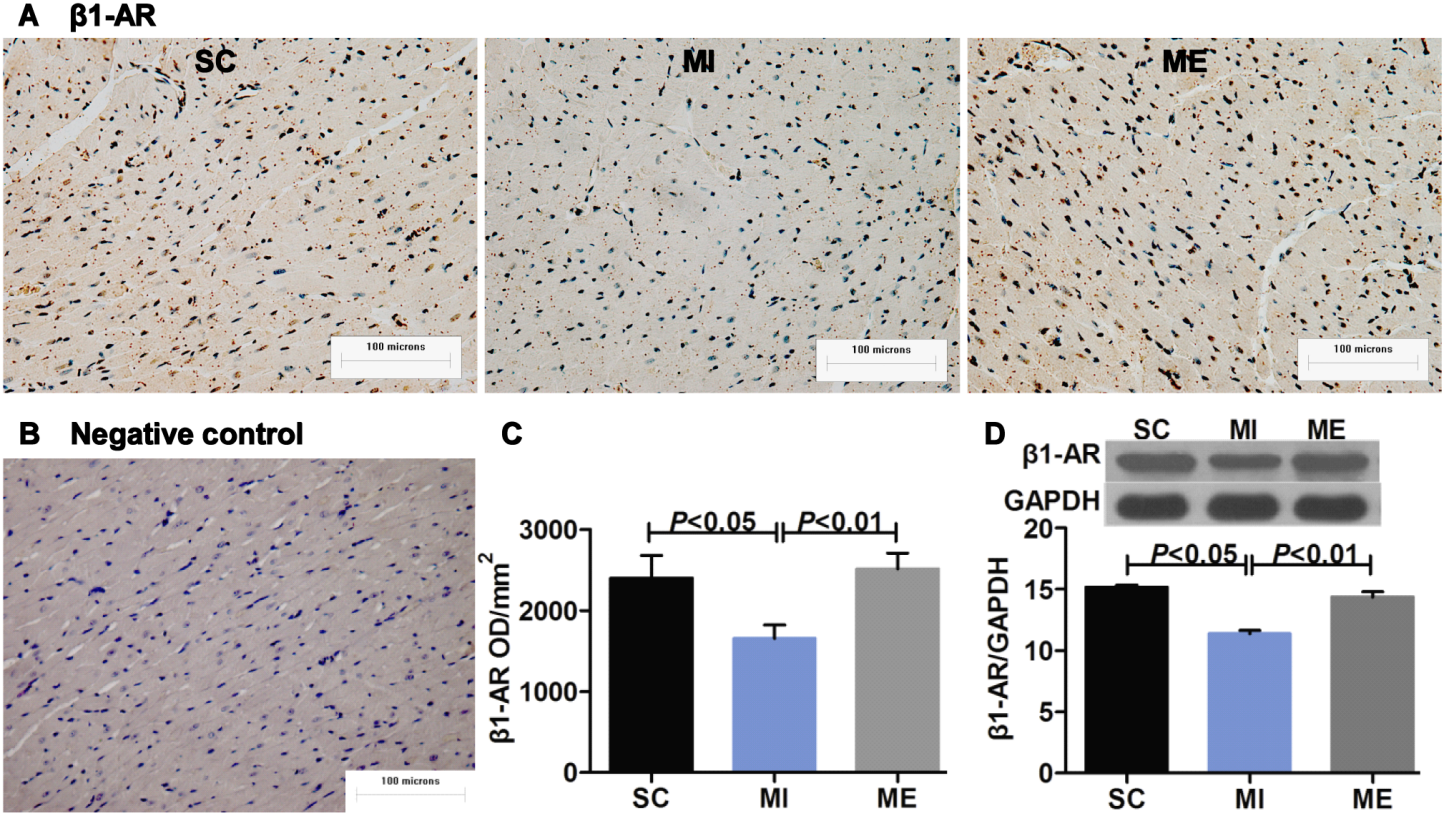
Effects of aerobic exercise on cardiac β1-AR after MI. A. Immunohistochemical staining of cardiac β1-AR. Scale bar = 100 microns. B. Negative staining control. C–D. The expression of cardiac β1-AR in the SC, MI, ME groups. Immunohistochemical staining and Western blot analysis indicated that the protein expression of β1-AR in the left ventricle was significantly reduced after MI. Aerobic exercise was able to increase the expression of β1-AR.

**Figure 5:**
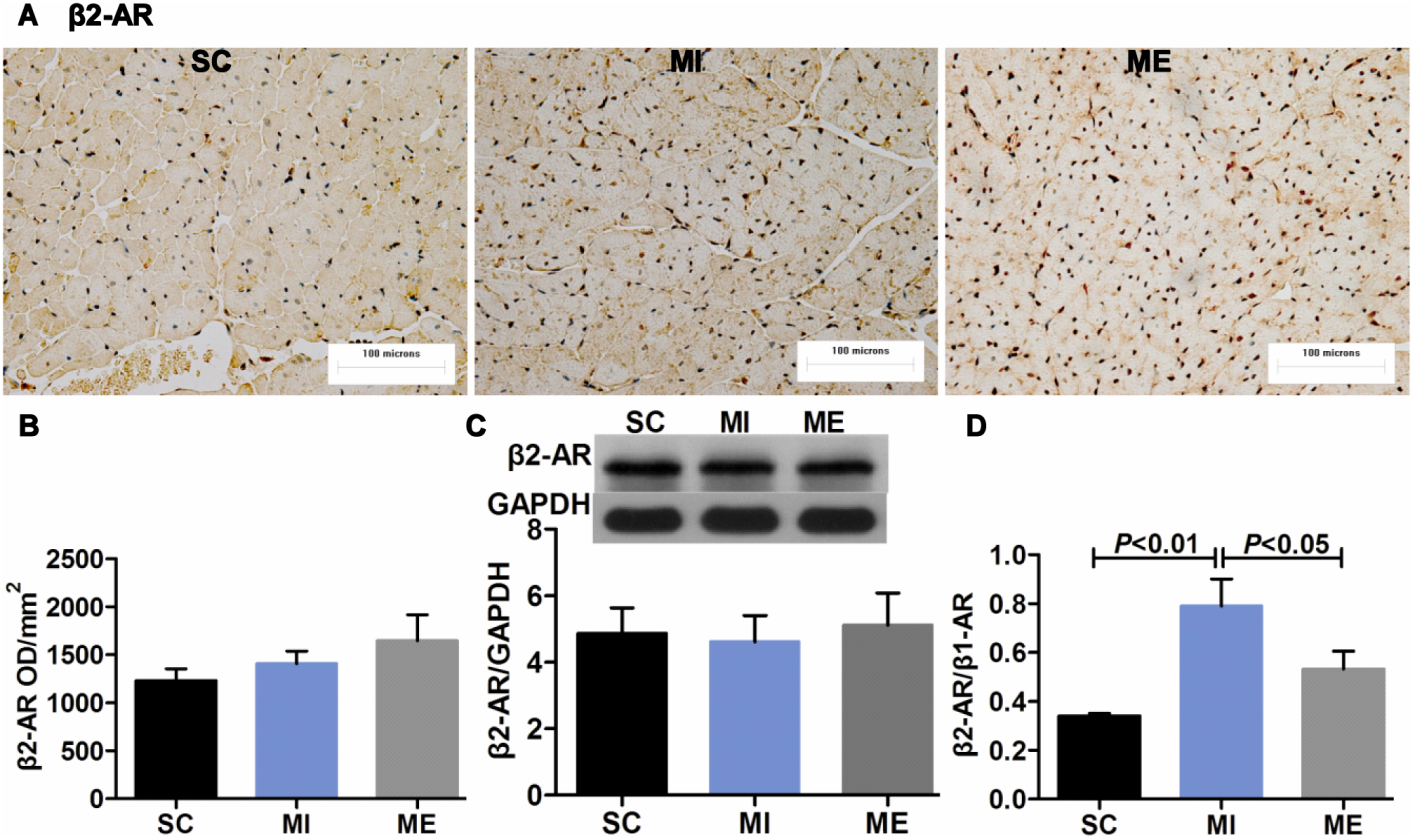
Effects of aerobic exercise on cardiac β2-AR after MI. A. Immunohistochemical staining of cardiac β2-AR. Scale bar = 100 microns. B–C. The expression of cardiac β2-AR in the SC, MI, ME groups. Immunohistochemical staining and Western blot analysis indicated that the expression of β2-AR remained unaltered in the SC, MI, ME group. D. The ratio of β2-AR/β1-AR. MI resulted in an increased ratio of β2-AR/β1-AR, and aerobic exercise normalized the ratio.

**Figure 6:**
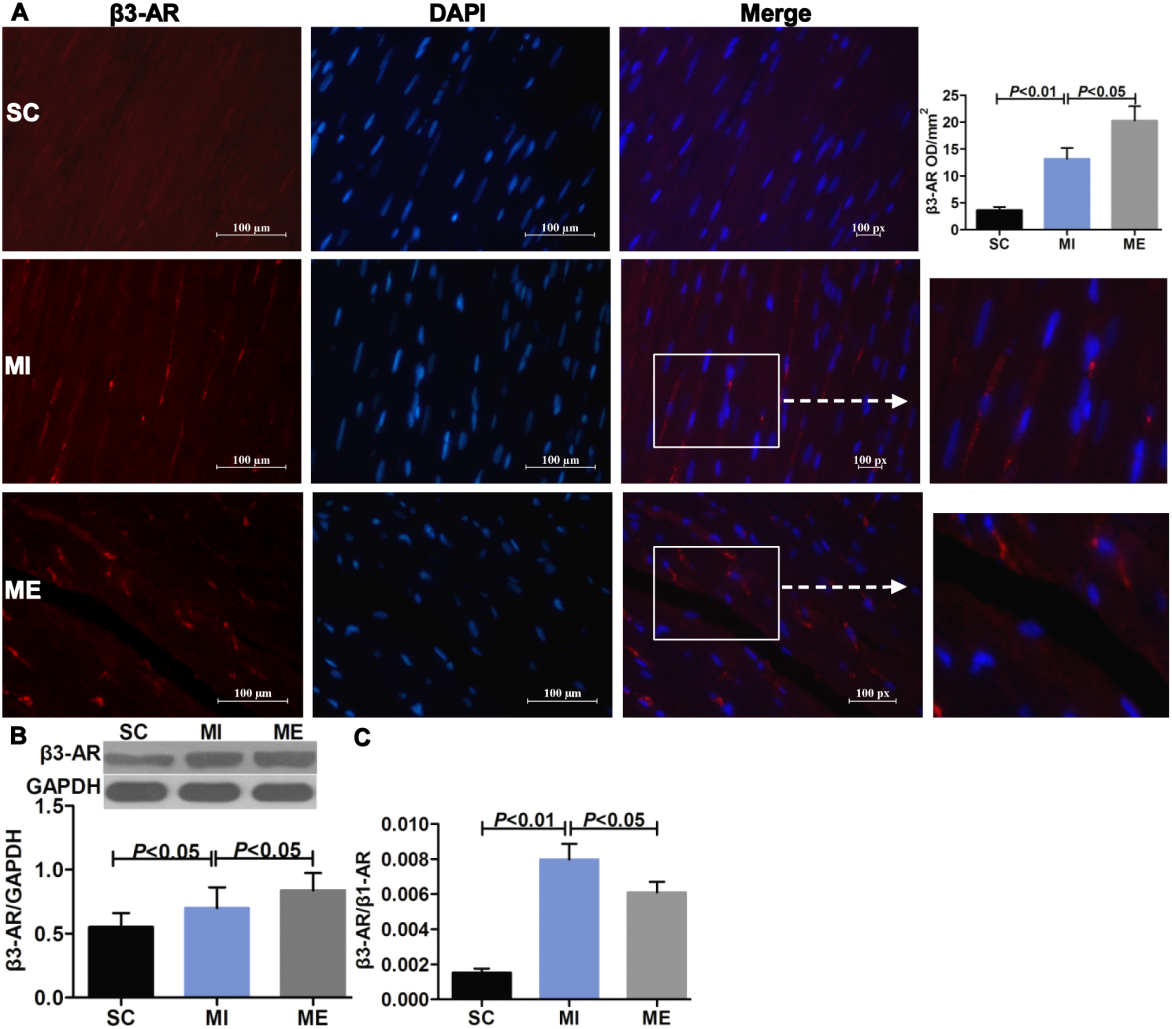
Effects of aerobic exercise on cardiac β3-AR after MI. A. Immunofluoresence staining of cardiac β3-AR (red fluorescence), the nuclei was labelled by DAPI (blue fluorescence). Scale bar = 100 microns. B. Western blot analysis of cardiac β3-AR in the SC, MI, ME groups. Immunofluoresence staining and Western blot analysis showed that cardiac β3-AR protein expression was significantly higher in the ME group compared with the SC and MI groups. C. The ratio of β3-AR/β1-AR. MI was associated with a significant increase in the ratio of β3-AR/β1-AR. Aerobic exercise was able to normalize the β3-AR/β1-AR ratio.

Compared with the SC group, the mean OD value for β1-AR (*F*_2,16_ = 6.11, *P* = 0.006) in the LV was reduced after MI (*P*<0.05) (**Figure 4A, C**). Furthermore, the protein expression of β1-AR after MI was significantly down-regulated (*P*<0.01 vs. SC) (**Figure 4D**). Whereas β2-AR (*F*_2,16_ = 1.13, *P* = 0.343) was both unaffected after MI in IHC staining and Western blot analysis (**Figure 5A, B, C**). The mean OD value of β3-AR (*F*_2,16_ = 15.55, *P*<0.001) was significantly increased after MI (*P*<0.01 vs. SC) (**Figure 6A**). Moreover, β3-AR protein (*F*_2,16_ = 9.66, *P* = 0.001) expression was up-regulated (*P*<0.05 vs. SC) following MI (**Figure 6B**). However, after 8 weeks of aerobic exercise, the OD value of β1-AR was increased compared with the MI group (*P*<0.01). This finding was supported by Western blot analysis of β1-AR, which indicated that the protein expression of β1-AR (*F*_2,16_ = 6.65, *P* = 0.005) was significantly up-regulated after aerobic exercise (*P*<0.01 vs. MI) (**Figure 4A, C, D**). And β2-AR (*F*_2,16_ = 1.76, *P* = 0.200) was unaltered after aerobic exercise (**Figure 5A, B, C**). The OD value of β3-AR was significantly increased after aerobic exercise compared with the MI group (*P*<0.05). Furthermore, β3-AR protein expression was up-regulated (*P*<0.05 vs. MI) following 8 weeks of aerobic exercise (**Figure 6A, B**). As expected, MI was associated with a significant increase in the ratios of β2-AR/β1-AR (*F*_2,15_ = 5.71, *P* = 0.014) (**Figure 5D**) and β3-AR/β1-AR (*F*_2,16_ = 19.08, *P*<0.001) (both *P*<0.01 vs. SC) (**Figure 6C**). Importantly, aerobic exercise was able to normalize the β2-AR/β1-AR, and β3-AR/β1-AR ratios (both *P*<0.05 vs. MI). The negative control images of IHC and IFC staining were provided (**Figure 4B**, **S2**).

**Figure 7:**
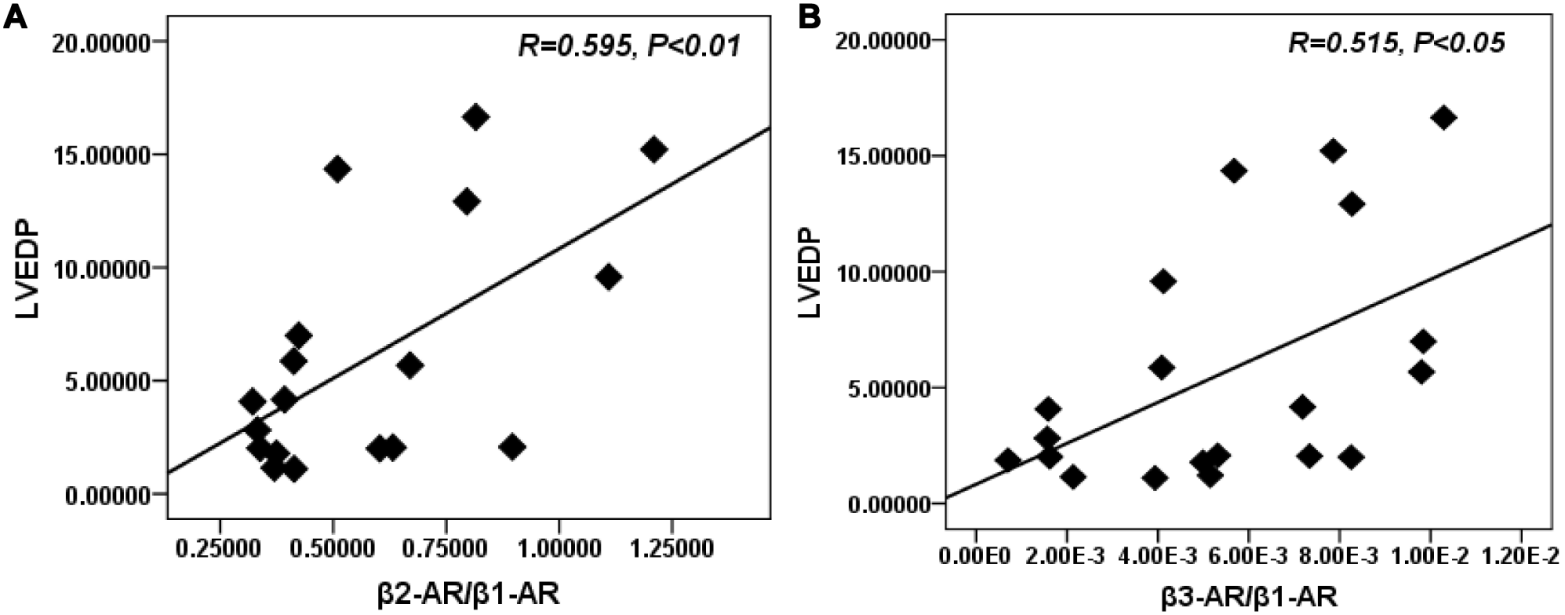
Correlation between LVEDP and the ratios of β-ARs. A. Positive correlation between LVEDP and β2-AR/β1-AR ratio. B. Positive correlation between LVEDP and β3-AR/β1-AR ratio. Coefficients of correlation (R) are indicated. There was a close correlation between the β-AR balance and hemodynamic parameter.

As shown in Figure 7, both β2-AR/β1-AR and β3-AR/β1-AR ratios were positively correlated with LVEDP, (R = 0.595, *P* = 0.005) (**Figure 7A**) (R = 0.515, *P* = 0.01) (**Figure 7B**), suggesting that the β-AR balance was closely correlated with hemodynamic parameter.

### Aerobic Exercise Increases the Expression of β3-AR after MI through the Activation of NOS2 and NOS1

**Figure 8:**
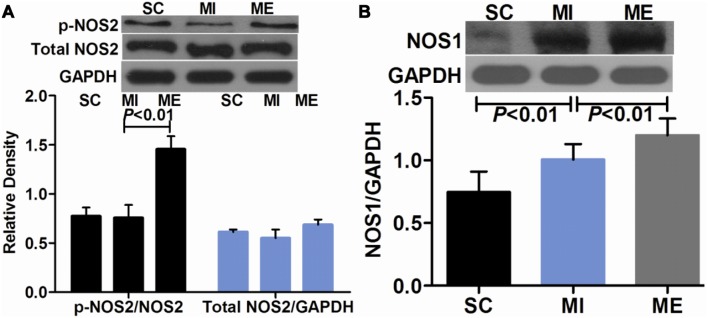
Effects of aerobic exercise on cardiac NOS2 and NOS1 expression after MI. A. Western blot analysis of cardiac NOS2 and p-NOS2 in the SC, MI, ME groups. The expression of total NOS2 and p-NOS2 remained constant in the SC and MI groups. Compared with the MI group, NOS2 was significantly activated in the ME group. B. Western blot analysis of cardiac NOS1 in the SC, MI, ME groups. The protein expression of NOS1 was significantly higher in the ME group compared with the SC and MI groups.

In this experiment, we measured 2 of the 3 major NOS isoforms (NOS2 and NOS1). As shown in Figure 8, compared with the SC group, the protein expression of NOS1 (*F*_2,16_ = 33.91, *P*<0.001) in the LV was significantly up-regulated after MI (*P*<0.01), while the expression of total NOS2 (*F*_2,16_ = 1.47, *P* = 0.251) and p-NOS2 (*F*_2,16_ = 11.30, *P* = 0.001) remained constant in the MI group. Compared with the MI group, aerobic exercise significantly increased the expression of NOS1 (*P*<0.01). Furthermore, NOS2 was activated through Ser1177 phosphorylation (*P*<0.01 vs. MI) by exercise in the failing heart, whereas the expression of total NOS2 was unaltered (**Figure 8**). And there was a positive correlation between β3-AR and NOS2 (R = 0.437, *P* = 0.048), and β3-AR and NOS1 expression (R = 0.057, *P* = 0.004) (**Figure S3**).

## Discussion

The major findings of the present study are: (i) 8 weeks of aerobic exercise inhibits cardiac sympathetic nerve sprouting and restores β3-AR/β1-AR balance after MI (ii) Aerobic exercise increases the expression of β3-AR through the activation of NOS2 and NOS1 following MI.

Growing evidence indicates that exercise, started early after MI, can improve cardiac function [19]–[21], [24] by increasing maximal stroke volume, ejection fraction [31] and attenuating LV contractile deterioration [21]. This study confirms previous evidence showing that aerobic exercise is effective in reducing infarct size and myocardial interstitial fibrosis. Furthermore, exercise can attenuate the deterioration in cardiac function after MI. The mechanisms of beneficial effects of exercise described above may be associated with exercise-induced cardiomyocyte proliferation [32], [33] and angiogenesis [9], attenuated myocardial apoptosis [34], [35], and improved myofilament function [25], as well as restored intracellular calcium handling [19]. In this study, we hypothesized that aerobic exercise following MI could inhibit sympathetic nerve sprouting and restore the balance of β3-AR/β1-AR.

The conception of “cardiac nerve sprouting” was well described by Zhou et al. [2]. MI results in nerve injury, followed by cardiac nerve regeneration via sympathetic axon sprouting [1]. TH serves as a location marker for sympathetic nerves, and GAP43 is a marker of nerve sprouting [1]. Previous studies demonstrated that the densities of TH- and GAP43-positive nerves significantly increased in the MI group at 3 days, 1 week (peak), and 1 month [2]. This study confirmed previous evidence showing that cardiac TH and GAP43 protein expression significantly increased after MI, implying that sympathetic nerve sprouting in infarcted hearts was more excessive than that in normal hearts. Importantly, aerobic exercise was able to downregulate the protein expression of TH and GAP43 following MI, this suggests that aerobic exercise is effective in attenuating cardiac nerve sprouting. Although the precise mechanisms of nerve sprouting after MI remain unclear, it is known that NGF may play a key role in this pathological process. The overexpression of NGF in the heart induces sympathetic hyperinnervation [36], whereas the volume of the sympathetic ganglia is significantly reduced in NGF knockout mice [37]. In agreement with previous studies, the present study showed that NGF expression was significantly increased in the MI group. Noticeably, the level of NGF was significantly reduced by aerobic exercise after MI, which may contribute to the reduction of sympathetic fiber innervation. This implied that the effects of exercise on the inhibition of nerve sprouting after MI were related to the attenuated levels of NGF. It is well established that excessive nerve sprouting may suppress the functions of transient outward current and inward rectifier current [6], thereby leading to ventricular arrhythmias [24]. Accordingly, the resulting normalization of nerve sprouting by exercise may provide a therapy to prevent arrhythmias.

Previous studies have suggested that exercise can increase β1-AR protein (48%) [25] and mRNA levels [38], increase cAMP levels (36%) [25], and reduce β2-AR responsiveness [26] in the diseased heart. Additionally, Billman et al demonstrated that a more normal β1/β2-AR balance was restored by exercise in animals susceptible to sudden death [26], but the density of β1- and β2-AR was not measured in the study. In the current study, MI resulted in increased ratios of β2-AR/β1-AR and β3-AR/β1-AR. Importantly, after 8 weeks of exercise, the protein expression of cardiac β1-AR and β3-AR was increased, while β2-AR expression did not change, implying that the β2-AR/β1-AR and β3-AR/β1-AR ratios were correspondingly restored. This indicated that MI resulted in an imbalance between the expression of the three β-AR subtypes and that exercise could normalize the β-AR, particularly the β3-AR/β1-AR balance after MI. Previous studies have reported that the downregulation of β1-AR after MI may lead to less production of cAMP, which results in blunted cardiac contractile responses [9]. And the opposite changes in β1- and β3-AR expression and the imbalance between their inotropic influences may lead to progressive cardiac dysfunction in the failing heart [11]. Furthermore, the activation of β2-AR in the diseased heart [12] can increase the risk for arrhythmias [13], [26]. Accordingly, the resulting restoration of the β-AR balance by exercise may provide a therapy to prevent cardiac dysfunction.

In contrast to β1- and β2-AR, β3-AR modulates a negative inotropic effect through inhibitory G-protein coupled NOS/NO signaling [39]. After MI, β3-AR is activated by high concentrations of norepinephrine and is described as a counter-mechanism during sympathetic overstimulation [18]. Recently, β3-AR has been shown to play a protective role in the development of MI [14], [39], [40]. The stimulation of β3-AR blunts cardiac contractile responses and improves LV function in the failing heart [18]. Additionally, specific β3-AR agonists can protect the heart from cardiac hypertrophy through generating NO and reducing ROS [14], [16]. In this study, the data showed that compared with the MI group, exercise significantly increased the protein expression of β3-AR in the LV, implying that β3-AR may play a role in the beneficial effects of exercise. Although it is still unclear how exercise protects against MI, our data suggest that β3-AR is involved in this process. The upregulation of the β3-AR may trigger many cytoprotective signaling cascades, which ultimately contribute to the cardioprotection. In this study, our attention was focused on NOS2 and NOS1 in the heart. Previous studies suggested that NOS2 was solely responsible for β3-AR-induced NO production [41]. However, new research indicates that NOS1 also plays a key role in β3-AR signaling [16]. The importance of NOS1 to cardiac calcium cycling and contractility has been revealed in recent studies [18]. In the present study, NOS1 protein expression in the LV increased after MI. And exercise increased the expression of NOS1 and the activation of NOS2 without altering total NOS2, suggesting that exercise upregulates β3-AR expression, making β3-AR a possible source for NOS2 and NOS1 activation. Recent studies have shown that activation of NOS2 and NOS1 is essential for β3-AR-induced cardioprotection [16], [18], and the beneficial effects of β3-AR stimulation were lost in NOS2 and NOS1 knockout mice [42]. Importantly, NOS2 and NOS1 are indispensable for the cardiac adaptive effects of exercise [35], [43]. New data suggests that the beneficial effects of exercise are mediated by increased NOS1 signaling, which leads to increased cardiac calcium cycling, followed by enhanced contraction and accelerated relaxation [43]. Furthermore, exercise failed to produce any beneficial adaptations in NOS2 and NOS1 knockout mice [35], [43]. This suggests that the beneficial effects of the β3-AR stimulation after exercise may be associated with the activation of NOS2 and NOS1. Additionally, in this study, both β2-AR/β1-AR and β3-AR/β1-AR ratios were closely correlated with hemodynamics, this implied that the normalization of β-AR balance by exercise may be associated with improvement of cardiac function. Although the causality among these findings remains uncertain, it is possible that the activation of NOS2 and NOS1 is involved in this process.

### Study Limitations

Our study shows that aerobic exercise inhibits cardiac sympathetic nerve sprouting and restores β3-AR/β1-AR balance after MI. However, it remains unclear if this contributes to a benefit for cardiac function. Although an association among these findings has been described, it does not provide a direct evidence of cause-effect.

## Conclusions

In summary, the current study demonstrated that 8 weeks of aerobic exercise can improve cardiac function after MI and that the underlying mechanisms may be related to the inhibition of sympathetic nerve sprouting, the restoring of β3-AR/β1-AR balance, and the upregulation of β3-AR.

## Supporting Information

Figure S1 **Effects of aerobic exercise on electrocardiographic recording.** MI was recognized on an electrocardiogram by ST-segment elevation. MI resulted in an elevated ST-segment, which was attenuated by aerobic exercise.
(TIF)

Figure S2 **The negative staining control image of IFC.** No staining was observed in the negative control.
(TIF)

Figure S3 **Correlation between cardiac β3-adrenergic receptors and eNOS, nNOS expression.** A. Positive correlation between cardiac β3-AR and eNOS expression, B. Positive correlation between cardiac β3-AR and nNOS expression. Coefficients of correlation (R) are indicated.
(TIF)
