# Supplementary figures and images for: Aerobic Exercise Inhibits Sympathetic Nerve Sprouting and Restores β-Adrenergic Receptor Balance in Rats with Myocardial Infarction
Source: PLoS One. 2014 May 19;9(5):e97810. doi: 10.1371/journal.pone.0097810 (PMC4026473; doi:10.1371/journal.pone.0097810)

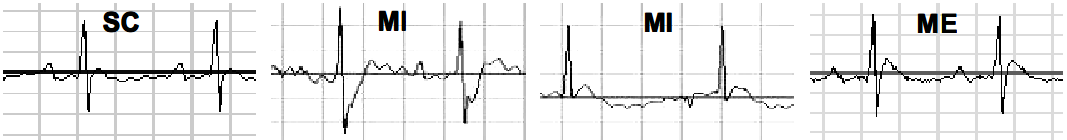

Supplement: Figure S1 — Effects of aerobic exercise on electrocardiographic recording. MI was recognized on an electrocardiogram by ST-segment elevation. MI resulted in an elevated ST-segment, which was attenuated by aerobic exercise. (TIF) [file pone.0097810.s001.tif]

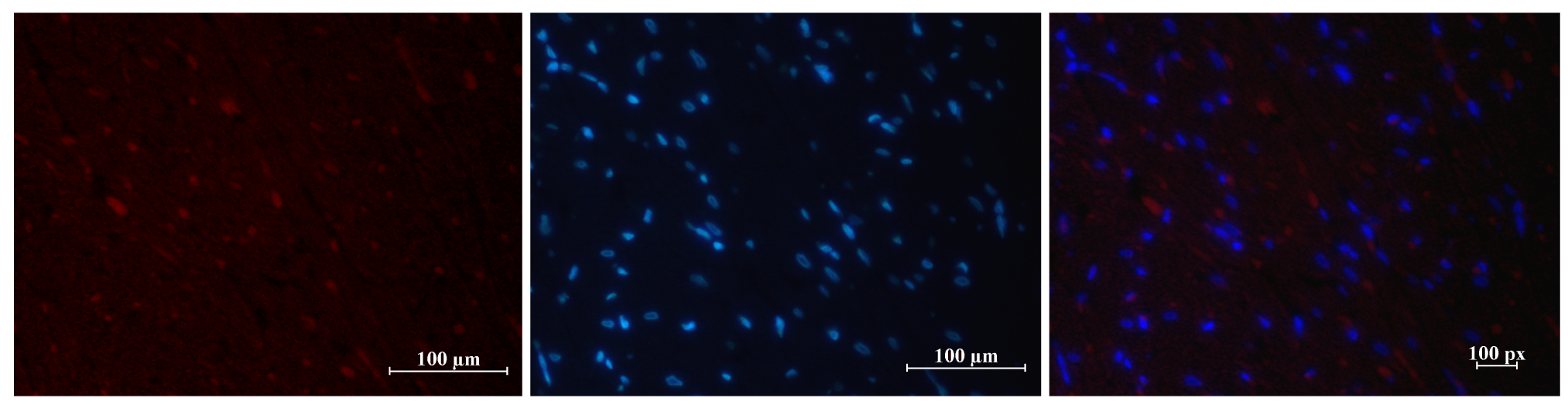

Supplement: Figure S2 — The negative staining control image of IFC. No staining was observed in the negative control. (TIF) [file pone.0097810.s002.tif]

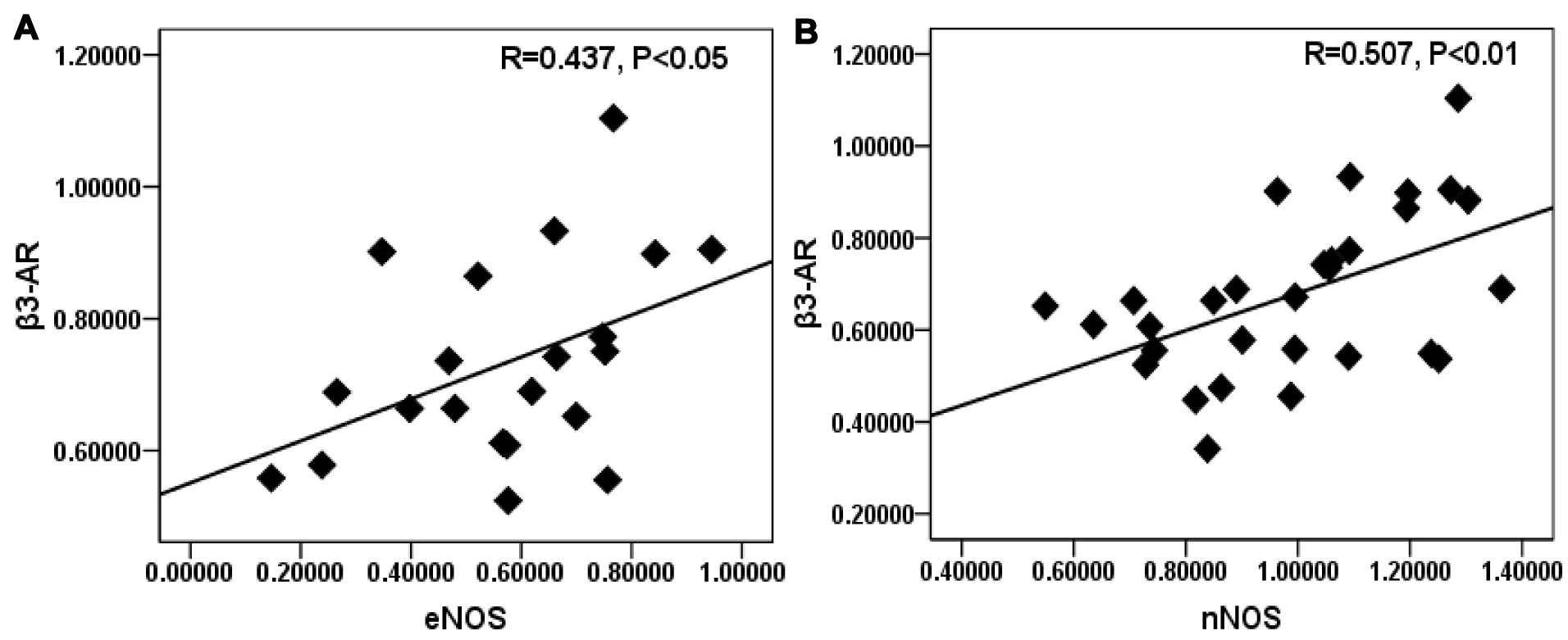

Supplement: Figure S3 — Correlation between cardiac β3-adrenergic receptors and eNOS, nNOS expression. A. Positive correlation between cardiac β3-AR and eNOS expression, B. Positive correlation between cardiac β3-AR and nNOS expression. Coefficients of correlation (R) are indicated. (TIF) [file pone.0097810.s003.tif]
